# Is it possible to introduce an interview to the Korean Medical Licensing Examination to assess professional attributes?: a survey-based observational study

**DOI:** 10.3352/jeehp.2022.19.10

**Published:** 2022-05-10

**Authors:** Seung-Joo Na, HyeRin Roh, Kyung Hee Chun, Kyung Hye Park, Do-Hwan Kim

**Affiliations:** 1 Department of Medical Education, CHA University School of Medicine, Pocheon, Korea; 2 Department of Medical Education, Inje University College of Medicine, Busan, Korea; 3 Department of Humanities & Social Sciences in Medicine, Inje University College of Medicine, Busan, Korea; 4 Institute for Medical Humanities, Inje University College of Medicine, Busan, Korea; 5 Department of Medical Education, Konyang University College of Medicine, Daejeon, Korea; 6 Department of Emergency Medicine, Yonsei Wonju Christian Hospital, Wonju, Korea; 7 Department of Medical Education, Yonsei University Wonju College of Medicine, Wonju, Korea; 8 Department of Medical Education, Hanyang University College of Medicine, Seoul, Korea; Hallym University, Korea

**Keywords:** Medical education, Medical licensure, Professionalism, Professional competence, Republic of Korea

## Abstract

**Purpose:**

This study aimed to gather opinions from medical educators on the possibility of introducing an interview to the Korean Medical Licensing Examination (KMLE) to assess professional attributes. Specifically following topics were dealt with: the appropriate timing and tool to assess unprofessional conduct; the possiblity of prevention of unprofessional conduct by introducing an interview to the KMLE; and the possibility of implementation of an interview to the KMLE.

**Methods:**

A cross-sectional study approach based on a survey questionnaire was adopted. We analyzed 104 pieces of news about doctors’ unprofessional conduct to determine the deficient professional attributes. We derived 24 items of unprofessional conduct and developed the questionnaire and surveyed 250 members of the Korean Society of Medical Education 2 times. Descriptive statistics, cross-tabulation analysis, and Fisher’s exact test were applied to the responses. The answers to the open-ended questions were analyzed using conventional content analysis.

**Results:**

In the survey, 49 members (19.6%) responded. Out of 49, 24 (49.5%) responded in the 2nd survey. To assess unprofessional conduct, there was no dominant timing among basic medical education (BME), KMLE, and continuing professional development (CPD). There was no overwhelming assessment tool among written examination, objective structured clinical examination, practice observation, and interview. Response rates of “impossible” (49.0%) and “possible” (42.9%) suggested an interview of the KMLE prevented unprofessional conduct. In terms of implementation, “impossible” (50.0%) was selected more often than “possible” (33.3%).

**Conclusion:**

Professional attributes should be assessed by various tools over the period from BME to CPD. Hence, it may be impossible to introduce an interview to assess professional attributes to the KMLE, and a system is needed such as self-regulation by the professional body rather than licensing examination.

## Introduction

### Background/rationale

In recent years in Korea, the media has often exposed the unprofessional conduct of doctors. Korean doctors are allowed to practice after medical school graduation and pass the Korean Medical Licensing Examination (KMLE), and they can practice for a lifetime after completing the continuing medical education for at least 8 hours a year. The public demanded that assessing the humanity or virtue of doctors be added to the KMLE to prevent the unprofessional conduct of doctors. Even a member of the National Assembly insisted on the need to introduce an interview to the KMLE [1]. Literature shows that humanity or virtue is hard to assess, especially as a single examination [2,3]. However, professional attributes can be taught and evaluated continuously [4-7]. Therefore, medical educators focused on professional conduct or attributes [4,5]. Furthermore, to the best of our knowledge, there are no reports of an interview in the licensing exam in the literature from PubMed, Web of Science Core Collection, Scopus, and KoreaMed.

### Objectives

This study seeks to gather opinions from medical educators regarding the possibility of assessing professional attributes through an interview in the KMLE. Specific research questions are (1) the appropriate timing, (2) the appropriate tool to assess unprofessional conduct, (3) the possibility of an interview to prevent future unprofessional conduct, and (4) the possibility of implementing an interview to assess professional attributes in the KMLE.

## Methods

### Ethics statement

This study was approved by the Institutional Review Board of Inje University (approval no., 118.038.025.149). Informed consent was obtained from participants.

### Study design

This was a survey-based observational study. It was described according to the STROBE (Strengthening the Reporting of Observational Studies in Epidemiology) Statement (https://www.strobe-statement.org/).

### Setting

The survey was conducted for 2 months, from February to March 2020. The survey was sent twice, and it was conducted via e-mail. In the second e-mail survey, we added one question about the possibility of implementing an interview to assess professional attributes in the KMLE.

### Participants

The survey subjects were 250 members (as of December 2019) of the Korean Society of Medical Education. The first survey respondents were 57, and the second survey respondents were 24. The 37 responses that did not respond to appropriate timing or tool to assess unprofessional conduct were excluded.

### Variables

The 4 main variables of interest were (1) the appropriate timing, (2) the appropriate assessment tool to prevent the 24 items defined as unprofessional conduct, (3) the possibility of assessment to prevent future unprofessional conduct, and (4) the possibility of implementing an interview to assess professional attributes in the KMLE. The appropriate timing options were the basic medical education (BME) period, in the KMLE, graduate medical education (GME), and continuing professional development (CPD) period. The assessment tool options were written examination, objective structured clinical examination (OSCE), practice observation, and interview.

### Data sources/measurement

Before the survey, we analyzed 104 pieces of news published from 2011 to 2019 about doctors’ unprofessional conduct using the Critical Incident Technique (CIT) to determine what the public thinks doctors lack in their professional attributes. CIT is one of the qualitative research methods and refers to collecting direct observational information on human behavior, the circumstances, and the contents of critical incidents for use in problem-solving [8,9].

We categorized 104 news into 24 items of unprofessional conduct of doctors. Based on these categories, we developed a survey questionnaire consisting of 26 nominal scales and 3 open-ended questions. A total of 2 questions asked the reason for the responses and 1 free opinion. To secure content validity, 5 medical educationalists, including medical doctors, developed questionnaires and critically reviewed questionnaires 4 times. No reliability test was conducted due to the descriptive characteristics of the survey tool. A survey questionnaire is presented in Supplement 1.

### Bias

To avoid potential sources of bias, we selected all regular members of the Korean Society of Medical Education as respondents. We checked whether there was any discriminatory terminology in 24 types of unprofessional conduct reflected in the questionnaire.

### Study size

Since it is not a controlled study, the study size estimation was not possible. Only those who agreed to participate voluntarily were included.

### Statistical methods

Descriptive statistics, a cross-tabulation analysis, and Fisher’s exact test were applied to the questionnaire responses using the JAMOVI ver. 2.2.2 program (https://www.jamovi.org). As the expected frequency of fewer than 5 cells accounted for more than 20% of the total cells, the significance probability was confirmed by Fisher’s exact test. The statistical significance was set at 0.05. We analyzed the responses to the open-ended questions using the conventional content analysis method [10]. According to the method, we categorized the responses into keywords. The primary coding and category classification was done by the first author (S.J.N.), and all researchers reviewed the classification content through 3 meetings to ensure content validity.

## Results

### Participants

A total of 49 respondents were the subject of the final analysis. Of these, 24 answered the added question of the second survey. Detailed demographic information about the 49 respondents is reported in Table 1. Raw responses data of two surveys are available from Dataset 1.

### Appropriate timing of the assessment to prevent the 24 items of unprofessional conduct

The appropriate timing required to assess 24 items of unprofessional conduct of doctors is presented in Table 2. None of the appropriate timings had an overwhelming response rate for assessing specific unprofessional conduct, except for 2 items. “In the BME” was the dominant timing for 2 items “posting patients’ information on social media” (61.2%), and “sexual assault in the healthcare environment” (55.1%). The average number of respondents who answered was 18.3 (37.4%) in the BME, 15.2 (31.0%) in GME/CPD, 11.5 (23.6%) in the KMLE, and 3.9 (8.0%) suggested no assessment required.

### An appropriate assessment tool to prevent the 24 items of unprofessional conduct

The appropriate assessment tools to prevent the 24 items of unprofessional conduct are presented in Table 3. None of the 4 assessment tools had an overwhelming response rate for assessing specific unprofessional conduct. An average of 15.3 (35.9%) respondents selected a written exam, 14.7 (34.4%) practice observation, 8.1 (19.1%) an interview, and 4.5 (10.6%) OSCE.

### Possibility of preventing unprofessional conduct when an interview is introduced to the KMLE

The responses to whether introducing an interview to the KMLE could prevent unprofessional conduct are provided in Table 4. There were slightly more “impossible” (n=24, 49.0%) responses than “possible” (n=21, 42.9%). A total of 24 (63.2%) out of 38 medical educators with more than 5 years of experience answered “impossible.” A total of 4 (80.0%) out of 5 medical educators with less than 5 years of experience answered “possible.” There was a statistically significant difference according to the medical education experience (P=0.048).

The respondents’ descriptions of the reasons for their thoughts/opinions are presented in Table 5. Of the multiple options, 22 respondents responded “impossible” to 27 items. Those who stated “impossible” said that the examination could not predict unprofessional conduct (n=11, 40.7%), that professional attributes were difficult to measure by licensing examination (n=9, 23.3%), should be assessed continuously from BME to CPD (n=4, 14.8%), and an interview was inappropriate as an assessment tool (n=3, 11.1%).

Of the multiple options, 19 respondents responded “possible” to 19 items. The overwhelming response was an impact of emphasizing professional attributes in BME (n=21, 91.3%). There were also responses that an interview was appropriate as an assessment tool (n=2, 8.7%).

### Possibility of implementing an interview to assess professional attributes in the KMLE

The responses regarding whether implementing an interview in the KMLE was possible are presented in Table 6. “Impossible” (n=12, 50.0%) was selected more often than “possible” (n=8, 33.3%). There was no statistically significant difference between groups according to demographic characteristics.

The contents of the respondents’ descriptions of the reasons for their thoughts/opinions are provided in Table 7. Respondents who “impossible” said that ensuring the validity, reliability, and objectivity of the interview was difficult (n=5, 50.0%), there was no cost-effectiveness (n=4, 40.0%), and developing assessment questions were difficult (n=1, 10.0%). All respondents who answered “possible” said that implementing an interview depends on the willingness of the examination institution (n=2, 100%).

## Discussion

### Key results

Medical educators responded evenly to the BME, the KMLE, and GME/CPD at appropriate assessment timing to assess preventing the unprofessional conduct of doctors. Medical educators did not overwhelmingly select an appropriate assessment tool during the written examination, practice observation, an interview, and OSCE. Medical educators responded with “impossible” and “possible” at a similar rate to prevent unprofessional conduct by introducing an interview to the KMLE. However, half of the medical educators answered “impossible” for implementing an interview in the KMLE, and “possible” was even lower.

### Interpretation

There was no overwhelming timing to assess doctors’ unprofessional conduct, which may imply that the professional conduct should be continuously assessed during the BME, in the KMLE, and GME/CPD period, not a specific period. This is because professional attributes are not acquired at a specific time but require continuous development. Therefore, the public’s argument that assessing professional attributes in a single test, such as licensing examination, can screen unprofessional doctors does not seem valid.

There was no dominant assessment tool among the 4 assessment tools, which may imply that various tools should be used instead of one specific tool to assess professional attributes. The appropriate tools are different depending on the professional attributes to be evaluated, and using multiple tools, not just one, is important for accurate measurement. This result can be interpreted as not valid for the public argument that an interview is the best tool for assessing professional attributes. Rather than blaming the unprofessional conduct of individual doctors, it seems more appropriate to create a system that continuously educates and assesses professional performance. Therefore, instead of enabling practice throughout a lifetime with a single test such as licensing examination, professional bodies should continuously assess colleagues to ensure they are not engaged in unprofessional conduct.

The response rates of “impossible” and “possible” to prevent unprofessional conduct by introducing an interview in the KMLE were similar. The position of “impossible” focused on whether professional attributes were measurable in the licensing examination and whether an interview could successfully discriminate as an assessment tool. However, the “possible” position seemed to focus on the educational effect that professional attributes are taught as examination content. There was a difference in that “impossible” was selected from a psychometric perspective and “possible” from an educational perspective. It is interesting to note that both the positions of “impossible” and “possible” posit that professional attributes should be important education content in the BME. However, it is known that a downside is that assessment drives learning. There is a risk that medical students focus on the skills they need to achieve high scores. The BME is a very important period in which medical students can develop their professional identity. However, education and assessment in GME/CPD are also emphasized as doctors need to be professional in practice.

Regarding the implementation of an interview to assess professional attributes in the KMLE, “impossible” was selected more than “possible.” The position of “impossible” seemed to consider practical aspects such as questions (or scenario) development, rater recruitment, and rater training for quality control of an interview. The position of “possible” considered policy perspectives such as the willingness of licensing examination administration. However, it seems impossible to develop questions and recruit and train raters to implement interviews with more than 3,000 examinees, only with the willingness of the examination institution.

In Korea, some unprofessional conduct of doctors is subject to criminal punishment. A change is needed to a system that allows doctors to self-regulate as a professional group rather than legal punishment. Therefore, a system should be developed in which professional bodies regulate doctors autonomously.

### Comparison with previous studies

Previous studies argued that professional attributes are not acquired all at once, so should be taught and assessed longitudinally even after graduating from medical school, and self-regulation should be implemented as a lifelong practice [4,5].

In meta-analysis studies on professionalism measures, it is necessary to use an appropriate assessment tool for the purpose and target of the assessment [6,7]. Various tools such as self-assessment, direct observation, peer assessment, patients’ opinions, and role model evaluation have been developed and used to measure professional attributes [6].

Professional attributes include affective domains such as attitudes, values, and goals [4]. According to previous studies, the assessment of the affective domain is less reliable than the cognitive domain [3], and thus should be continuously assessed with various tools [4].

In foreign countries such as the Unties States, Canada, United Kingdom, and Australia, professional bodies manage medical licensure, and licenses are renewed according to workplace-based assessment results every 1 to 5 years [11]. The public or colleagues may report members’ unprofessional conduct to professional bodies, and these warn or discipline members according to the results of their own investigation [12,13].

### Limitations

This study has a limitation in that the number of medical educators who responded to the survey was small.

### Suggestions

In future studies, an academic review from a psychometric perspective is necessary to introduce interviews to assess professional attributes in the medical licensing examination.

### Conclusion

More participants said that the introduction of interviews in licensing examinations cannot prevent the unprofessional conduct of doctors, and has no cost-effectiveness. Professional attributes should be continuously taught and assessed over the period from BME to CPD rather than a single test such as licensing examination. However, unprofessional attributes cannot be prevented by education and assessment. Therefore, a system is needed for recertification of medical licenses and self-regulation by the professional body.

## Figures and Tables

**Table 1.** Respondents’ current state of affairs

Characteristic	No. (%)
All	49 (100.0)
Gender	
Man	32 (65.3)
Woman	17 (34.7)
Medical license	
Yes	40 (81.6)
No	9 (18.4)
Affiliation	
Basic medical sciences	6 (12.2)
Clinical sciences	22 (44.9)
Social sciences and/or medical humanities	2 (4.1)
Medical education	17 (34.7)
Others	2 (4.1)
Position	
Professor	47 (95.9)
Teaching assistant or researcher	1 (2.0)
Others	1 (2.00
Experience in medical education	
Less than 1 year	-
Over 1 year–less than 5 years	5 (10.2)
Over 5 years–less than 10 years	6 (12.2)
Over 10 years	38 (77.6)
Educational activities (multiple responses)	
Lecture	47 (95.9)
Preceptor in clinical clerkship	28 (57.1)
Clinical skills laboratories	25 (51.0)
Supervision of resident	28 (57.1)
Continuing professional development	17 (34.7)
Others	7 (14.3)
Assessment activities (multiple responses)	
Admission selection	32 (65.3)
Written exam	32 (65.3)
Workplace-based assessment in clinical clerkship	27 (55.1)
Clinical skills assessment	25 (51.0)
Portfolio	20 (40.8)
Assessment of specialist	20 (40.8)
Others	1 (2.0)
Experience in the Korean Medical Licensing Examination/the certifying and qualifying examination for specialist	
Yes	31 (63.3)
No	18 (36.7)

**Table 2.** Appropriate assessment time for each unprofessional conduct (N=49)

Unprofessional conduct	BME	KMLE	GME/CPD	No assessment is required.
Concealing medical malpractice	18 (36.7)	10 (20.4)	18 (36.7)	3 (6.1)
Divulging medical information	18 (36.7)	15 (30.6)	13 (26.5)	3 (6.1)
Falling accidents in the hospital	23 (46.9)	8 (16.3)	13 (26.5)	5 (10.2)
False recording	20 (40.8)	16 (32.7)	9 (18.4)	4 (8.2)
Foreign body retention	14 (28.6)	9 (18.4)	22 (44.9)	4 (8.2)
Ghost surgery/treatment	12 (24.5)	14 (28.6)	20 (40.8)	3 (6.1)
Issuing fake medical documents/death certificate	17 (34.7)	17 (34.7)	12 (24.5)	3 (6.1)
Lending a license to an unqualified person	12 (24.5)	19 (38.8)	15 (30.6)	3 (6.1)
Medical negligence/malpractice	12 (24.5)	10 (20.4)	23 (46.9)	4 (8.2)
Medication errors	17 (34.7)	12 (24.5)	15 (30.6)	5 (10.2)
Misuse of propofol/psychotropic substances	16 (32.7)	6 (12.2)	21 (42.9)	6 (12.2)
Non-identification of a patient	18 (36.7)	15 (30.6)	12 (24.5)	4 (8.2)
Operation/treatment without informed consent	24 (49.0)	15 (30.6)	8 (16.3)	2 (4.1)
Posting patients’ information on social media	30 (61.2)	10 (20.4)	5 (10.2)	4 (8.2)
Practice or operation in a drunken state	22 (44.9)	8 (16.3)	15 (30.6)	4 (8.2)
Procedures without preparation for unanticipated events	13 (26.5)	16 (32.7)	16 (32.7)	4 (8.2)
Repetitive use of disposable syringes	18 (36.7)	12 (24.5)	17 (34.7)	2 (4.1)
Sexual assault in the healthcare environment	27 (55.1)	4 (8.2)	11 (22.4)	7 (14.3)
Sexual harassment/assault/intercourse with a patient	19 (38.8)	7 (14.3)	18 (36.7)	5 (10.2)
Sharing injection	23 (46.9)	11 (22.4)	13 (26.5)	2 (4.1)
Taking bribes	17 (34.7)	11 (22.4)	17 (34.7)	4 (8.2)
Transfusion complications	19 (39.6)	18 (37.5)	8 (16.7)	3 (6.3)
Unevidenced treatment	11 (22.4)	10 (20.4)	24 (49.0)	4 (8.2)
Violence between health care workers	20 (40.8)	4 (8.2)	19 (38.8)	6 (12.2)
Average of total	18.3 (37.4)	11.5 (23.6)	15.2 (31.0)	3.9 (8.0)

Values are presented as number (%). BME, Basic Medical Education; KMLE, Korean Medical Licensing Examination; GME, graduate medical education; CPD, continuing professional development.

**Table 3.** Appropriate assessment tool for each unprofessional conduct

Unprofessional conduct	No.	Written exam	OSCE	Practice observation	Interview
Concealing medical malpractice	44	9 (20.5)	4 (9.1)	17 (38.6)	14 (31.8)
Divulging medical information	45	21 (46.7)	2 (4.4)	13 (28.9)	9 (20.0)
Falling accidents in the hospital	42	15 (35.7)	8 (19.0)	18 (42.9)	1 (2.4)
False recording	43	23 (53.5)	5 (11.6)	10 (23.3)	5 (11.6)
Foreign body retention	41	13 (31.7)	8 (19.5)	18 (43.9)	2 (4.9)
Ghost surgery/treatment	44	17 (38.6)	1 (2.3)	13 (29.5)	13 (29.5)
Issuing fake medical documents/death certificate	45	25 (55.6)	2 (4.4)	12 (26.7)	6 (13.3)
Lending a license to an unqualified person	42	24 (57.1)	3 (7.1)	5 (11.9)	10 (23.8)
Medical negligence/malpractice	43	11 (25.6)	4 (9.3)	20 (46.5)	8 (18.6)
Medication errors	42	14 (33.3)	9 (21.4)	15( 35.7)	4 (9.5)
Misuse of propofol/psychotropic substances	39	13 (33.3)	1 (2.6)	14 (35.9)	11 (28.2)
Non-identification of a patient	43	12 (27.9)	12 (27.9)	16 (37.2)	3 (7.0)
Operation/treatment without informed consent	45	14 (31.1)	9 (20.0)	16 (35.6)	6 (13.3)
Posting patients’ information on social media	43	22 (51.2)	-	15 (34.9)	6 (14.0)
Practice or operation in a drunken state	41	10 (24.4)	2 (4.9)	18 (43.9)	11 (26.8)
Procedures without preparation for unanticipated events	43	14 (32.6)	7 (16.3)	15 (34.9)	7 (16.3)
Repetitive use of disposable syringes	45	15 (33.3)	7 (15.6)	18 (40.0)	5 (11.1)
Sexual assault in the healthcare environment	41	8 (19.5)	1 (2.4)	17 (41.5)	15 (36.6)
Sexual harassment/assault/intercourse with a patient	41	15 (36.6)	-	13 (31.7)	13 (31.7)
Sharing injection	45	16 (35.6)	6 (13.3)	17 (37.8)	6 (13.3)
Taking bribes	42	17 (40.5)	1 (2.4)	10 (23.8)	14 (33.3)
Transfusion complications	43	17 (39.5)	15 (34.9)	10 (23.3)	1 (2.3)
Unevidenced treatment	41	15 (36.6)	1 (2.4)	15 (36.6)	10 (24.4)
Violence between health care workers	39	7 (17.9)	-	17 (43.6)	15 (38.5)
Average of total		15.3 (35.9)	4.5 (10.6)	14.7 (34.4)	8.1 (19.1)

Values are presented as number (%). OSCE, objective structured clinical examination.

**Table 4.** Possibility of preventing unprofessional conduct by the introduction of an interview to assess professional attributes

Variable	No.	Possible	Impossible	Not sure	P-value^a)^
All	49	21 (42.9)	24 (49.0)	4 (8.2)	-
Gender					1.000 (1.000)
Man	32	14 (43.8)	15 (46.9)	3 (9.4)	
Woman	17	7 (41.2)	9 (52.9)	1 (5.9)	
Medical license					0.182 (0.121)
Yes	41	15 (36.6)	22 (53.7)	4 (9.8)	
No	8	6 (75.0)	2 (25.0)	-	
Affiliation					0.729 (0.881)
Basic medical sciences	6	2 (33.3)	4 (66.7)	-	
Clinical sciences	22	10 (45.5)	10 (45.5)	2 (9.1)	
Social sciences and/or medical humanities	2	1 (50.0)	-	1 (50.0)	
Medical education	17	7 (41.2)	9 (52.9)	1 (5.9)	
Others	2	1 (50.0)	1 (50.0)	-	
Experience in medical education					0.072 (0.048^*^)
Over 1 year–less than 5	5	4 (80.0)	-	1 (20.0)	
Over 5 years–less than 10 years	6	1 (16.7)	5 (83.3)		
Over 10 years	38	16 (42.1)	19 (50.0)	3 (7.9)	
Experience in the Korean medical licensing/the certifying and qualifying examination for specialist					0.073 (0.073)
Yes	31	10 (32.3)	18 (58.1)	3 (9.7)	
No	18	11 (61.1)	6 (33.3)	1 (5.6)	

* P<0.05.

a) By Fisher’s exact test.

**Table 5.** Analysis of reasons for responses to the possibility of preventing unprofessional conduct

Category	Answer	No. (%)
Impossible (case=22, multiple responses)		27 (100.0)
Unpredictability of the examination		11 (40.7)
	An examination cannot predict unprofessional conduct.	6 (22.2)
	Examination and practice are different.	5 (18.5)
Difficulty to measure professional attributes in high-stakes examination		9 (23.3)
	Test takers may respond with the correct answer rather than an honest answer.	5 (18.5)
	Professional attributes cannot be measured with a single examination.	4 (14.8)
Importance of continuing assessment from the BME to the CPD		4 (14.8)
	Professional attributes should be taught and assessed during the BME.	3 (11.1)
	Professional conduct should be continuously assessed and feedback.	1 (3.7)
Inappropriate assessment tool		3 (11.1)
	Measure professional attributes are difficult/impossible by an interview.	2 (7.4)
	Assessments that observe standardized patient or real patient care are more appropriate.	1 (3.7)
Possible (case=19, multiple responses)		23 (100.0)
Educational impact on the BME due to examination preparation		21 (91.3)
	Medical students may be alerted by learning the relevant penalties for unprofessional conducts	8 (34.8)
	Professional attributes can be an important educational content in the BME.	7 (30.4)
	Introducing an interview into the KMLE itself helps prevent unprofessional conduct	5 (21.7)
	Professional attributes should be assessed before the KMLE	1 (4.3)
Interview as an appropriate assessment tool	An interview may screen test takers for unprofessional conduct.	2 (8.7)
Not sure (case=2)		2 (100.0)
Unpredictability of unprofessional conduct		2 (100.0)
	I am not sure whether professional attributes can be assessed adequately in the KMLE	1 (50.0)
	I am not sure because the assessment situation and the practice are different.	1 (50.0)

BME, Basic Medical Education; CPD, continuing professional development; KMLE, Korean Medical Licensing Examination.

**Table 6.** Possibility of implementing an interview to assess professional attributes in the Korean Medical Licensing Examination

Variable	No.	Possible	Impossible	Not sure	Other	P-value^a)^
All	24	8 (33.3)	12 (50.0)	3 (12.5)	1 (4.2)	
Gender						0.052
Man	18	6 (33.3)	11 (61.1)	1 (5.6)	-	
Woman	6	2 (33.3)	1 (16.7)	2 (33.3)	1 (16.7)	
Medical license						0.283
Yes	22	6 (27.3)	12 (54.5)	3 (13.6)	1 (4.5)	
No	2	2 (100.0).	-	-	-	
Affiliation						0.826
Basic medical sciences	3	-	3 (100.0)	-	-	
Clinical sciences	12	3 (25.0)	6 (50.0)	2 (16.7)	1 (8.3)	
Social sciences and/or medical humanities	2	1 (50.0)	1 (50.0)	-	-	
Medical education	6	3 (50.0)	2 (33.3)	1 (16.7)	-	
Others	1	1 (100.0)	-	-	-	
Experience in medical education						0.312
Over 1 year–less than 5	1	-	-	1 (100.0).	-	
Over 5 years–less than 10 years	1	-	1 (100.0).	-	-	
Over 10 years	22	8 (36.4)	11 (50.0)	2 (9.1)	1 (4.5)	
Experience in the Korean medical licensing/the certifying and qualifying examination for specialist						0.238
Yes	17	5 (29.4)	10 (58.8)	1 (5.9)	1 (5.9)	
No	7	3 (42.9)	2 (28.6)	2 (28.6)	-	

Values are presented as number (%).

a) By Fisher’s exact test.

**Table 7.** Analysis of response to the implementation of an interview to assess professional attributes in the Korean Medical Licensing Examination

Category	Answer	No. (%)
Impossible (case=10)		10 (100.0)
Difficulty in ensuring validity, reliability, and objectivity of interviews		5 (50.0)
	Because it is difficult to develop standardized interviews, objectivity cannot be ensured.	2 (20.0)
	It is difficult to ensure standardized raters.	2 (20.0)
	It is difficult to ensure the validity and reliability of the interviews.	1 (10.0)
No cost-effectiveness of the examination	Considering the input of human and material resources, the examination has no effect.	4 (40.0)
		
Difficulty in developing assessment questions	It is impossible to develop an accurate and fair assessment question.	1 (10.0)
Not sure or other (case=3)		3 (100.0)
Limitation of the assessment tool		2 (66.6)
	Recruiting many raters is difficult.	1 (33.3)
	Further research on assessment tools is needed.	1 (33.3)
Concerns about implementation according to plan	I do not know if it will be implemented depending on the purpose of introducing an interview.	1 (33.3)
Possible (case=2)		2 (100.0)
The willingness of the examination institution	If the Korea Health Personnel Licensing Examination Institute is willing to implement an interview, it is possible.	2 (100.0)

## Footnotes

**Data availability**
Data files are available from Harvard Dataverse: https://doi.org/10.7910/DVN/PWAWYL Dataset 1. Survey response data coding.

## Supplementary materials

Data files are available from Harvard Dataverse: https://doi.org/10.7910/DVN/PWAWYL

Supplement 1. Survey questionnaire.

Supplement 2. Audio recording of the abstract.

## References

[1] Yoon YC Medical licensing examination personality interview should be introduced to strengthen qualification screening. Medi:gate [Internet]. https://m.medigatenews.com/news/3721681061.

[2] Kwon DH, Kwon DH (2016). Educational evaluation.

[3] Kim MH (2015). Revisiting affective characteristics assessment for humanism education in school: recent trends in educational research. J Learn Cent Curric Instr.

[4] Chestnut DH (2017). On the road to professionalism. Anesthesiology.

[5] Mahajan R, Aruldhas BW, Sharma M, Badyal DK, Singh T (2016). Professionalism and ethics: a proposed curriculum for undergraduates. Int J Appl Basic Med Res.

[6] Li H, Ding N, Zhang Y, Liu Y, Wen D (2017). Assessing medical professionalism: a systematic review of instruments and their measurement properties. PLoS One.

[7] Tay KT, Ng S, Hee JM, Chia EW, Vythilingam D, Ong YT, Chiam M, Chin AM, Fong W, Wijaya L, Toh YP, Mason S, Krishna LK (2020). Assessing professionalism in medicine: a scoping review of assessment tools from 1990 to 2018. J Med Educ Curric Dev.

[8] FLANAGAN JC (1954). The critical incident technique. Psychol Bull.

[9] Gremler DD (2004). The critical incident technique in service research. J Serv Res.

[10] Hsieh HF, Shannon SE (2005). Three approaches to qualitative content analysis. Qual Health Res.

[11] Yim MK, Kim JK, Kim BH (2021). The medical license; focusing on the national examination system for granting a medical license in 8 countries including Korea [Internet]. https://vo.la/kcapfO.

[12] Wynia MK (2010). The role of professionalism and self-regulation in detecting impaired or incompetent physicians. JAMA.

[13] Lee E (2020). Analysis and implications of the regulation for health professionals in Ontario, Canada. Study Am Const Inst Am Const.

