# Supplementary material for: Is it possible to introduce an interview to the Korean Medical Licensing Examination to assess professional attributes?: a survey-based observational study
Source: J Educ Eval Health Prof. 2022 May 10;19:10. doi: 10.3352/jeehp.2022.19.10 (PMC9257525; doi:10.3352/jeehp.2022.19.10)
Supplement: Supplementary file 2 — Supplement 1. Survey questionnaire. [file jeehp-19-10-suppl.docx]

**Supplement 1.** Survey questionnaire

1. Please select the most appropriate assessment time and tool in your opinion, when conducting an assessment to prevent/early detect the unprofessional conduct of the doctors. Please select one option for each assessment timing and tool. (If you selected “No assessment required',” do not select the assessment tool.)

| **No.** | **Unprofessional conduct** | **Appropriate time of the assessment (choose only one)** | | | | **Appropriate assessment tool (choose only one)** | | | |
| --- | --- | --- | --- | --- | --- | --- | --- | --- | --- |
|  |  | During the BME | During the KMLE | During GME and CPD | No assessment required | Written exam | OSCE | Practical observation | Interview |
| 1 | Concealing medical malpractice |  |  |  |  |  |  |  |  |
| 2 | Divulging medical information |  |  |  |  |  |  |  |  |
| 3 | Falling accidents in the hospital |  |  |  |  |  |  |  |  |
| 4 | False recording |  |  |  |  |  |  |  |  |
| 5 | Foreign body retention |  |  |  |  |  |  |  |  |
| 6 | Ghost surgery/treatment |  |  |  |  |  |  |  |  |
| 7 | Issuing fake medical documents/death certificate |  |  |  |  |  |  |  |  |
| 8 | Lending a license to an unqualified person |  |  |  |  |  |  |  |  |
| 9 | Medical negligence/malpractice |  |  |  |  |  |  |  |  |
| 10 | Medication errors |  |  |  |  |  |  |  |  |
| 11 | Misuse of propofol/psychotropic substances |  |  |  |  |  |  |  |  |
| 12 | Non-identification of a patient |  |  |  |  |  |  |  |  |
| 13 | Operation/treatment without informed consent |  |  |  |  |  |  |  |  |
| 14 | Posting patients’ information on social media |  |  |  |  |  |  |  |  |
| 15 | Practice or operation in a drunken state |  |  |  |  |  |  |  |  |
| 16 | Procedures without preparation for unanticipated events |  |  |  |  |  |  |  |  |
| 17 | Repetitive use of disposable syringes |  |  |  |  |  |  |  |  |
| 18 | Sexual assault in the healthcare environment |  |  |  |  |  |  |  |  |
| 19 | Sexual harassment/assault/ intercourse with a patient |  |  |  |  |  |  |  |  |
| 20 | Sharing injection |  |  |  |  |  |  |  |  |
| 21 | Taking bribes |  |  |  |  |  |  |  |  |
| 22 | Transfusion complications |  |  |  |  |  |  |  |  |
| 23 | Unevidenced treatment |  |  |  |  |  |  |  |  |
| 24 | Violence between health care workers |  |  |  |  |  |  |  |  |

BME, basic medical education; KMLE, Korean Medical Licensing Examination; GME, graduate medical education; CPD, continuing professional development

2. Do you think that if an interview to assess professional attributes is introduced into the KMLE, will these 24 conducts be prevented?

□ Yes □ No □ Not sure

2-1. Please write the reason why you think so.

3. Do you think that it is possible to implement introducing an interview to assess professional attributes to the KMLE? (Added only in the 2nd survey.)

□ Yes □ No □ Not sure

3-1. Please write the reason why you think so. (Added only in the 2nd survey)

4. Please write your opinion about introducing the interview to assess professional attributes in KMLE.
